# Tolerogenic bone marrow-derived dendritic cells induce neuroprotective regulatory T cells in a model of Parkinson’s disease

**DOI:** 10.1186/s13024-018-0255-7

**Published:** 2018-05-21

**Authors:** Charles R. Schutt, Howard E. Gendelman, R. Lee Mosley

**Affiliations:** 1 Department of Pharmacology and Experimental Neuroscience, Center for Neurodegenerative Disorders, University of Nebraska Medical Center, Omaha, NE USA; 2 Department of Pharmacology and Experimental Neuroscience, University of Nebraska Medical Center, 985930 Nebraska Medical Center, Omaha, NE 68198-5930 USA

**Keywords:** Mice, Dendritic cells, DCs, Regulatory T cells, Tregs, MPTP, Parkinson’s disease, Neuroinflammation, Neurodegeneration

## Abstract

**Background:**

Administration of granulocyte-macrophage colony-stimulating factor (GM-CSF) increases regulatory T cell (Treg) number and function with control of neuroinflammation and neuronal protection in the 1-methyl-4-phenyl-1,2,3,6-tetrahydropyridine (MPTP) model of Parkinson’s disease (PD). Recently, we demonstrated in an early phase 1 clinical trial that GM-CSF also improves motor skills in PD patients. However, the mechanisms of Treg induction and its effects on neuroprotective responses remain unknown. As GM-CSF induces tolerogenic dendritic cells (DCs) that in turn convert conventional T cells to Tregs, the pathways for DC induction of Tregs were assessed.

**Methods:**

Following differentiation, bone marrow-derived dendritic cells (BMDCs) were cultured in media or GM-CSF with or without post-culture stimulation with nitrated α-synuclein (N-α-Syn). Expression of cell surface co-stimulatory molecules and proinflammatory cytokines, and induction of Tregs were evaluated. The neuroprotective capacity of tolerogenic BMDCs was assessed by adoptive transfer to MPTP-intoxicated mice. The extent of neuroinflammation and numbers of surviving dopaminergic neurons were assessed in relation to Treg numbers.

**Results:**

Co-culture of differentiated BMDCs with conventional T cells led to Treg induction. Stimulation of BMDCs with N-α-Syn increased expression of co-stimulatory molecules and proinflammatory cytokines, with modest increases in Treg numbers. In contrast, continued culture of BMDCs with GM-CSF modestly altered expression of co-stimulatory molecules and proinflammatory cytokines and chemokines, but decreased Treg induction. Continued culture in GM-CSF and combined stimulation with N-α-Syn reduced Treg induction to the lowest levels. Adoptive transfer of tolerogenic BMDCs to MPTP-intoxicated mice increased splenic Tregs, attenuated neuroinflammatory responses, and protected nigrostriatal dopaminergic neurons.

**Conclusions:**

GM-CSF acts broadly to differentiate DCs and affect immune transformation from effector to regulatory immune responses. DCs skew such immune responses by increasing Treg numbers and activities that serve to attenuate proinflammatory responses and augment neuroprotection.

## Background

Parkinson’s disease (PD) is the most common neurodegenerative movement disorder and is caused by degeneration of dopaminergic neurons in the substantia nigra pars compacta. Loss of these neurons, their projections to the striatum, and dopamine neurotransmitter leads to resting tremor, postural instability, bradykinesia, and rigidity [1]. While a minority of PD cases result from defined mutations that initiate PD, the majority of cases result from unknown events [2].

Both, the innate and adaptive arms of the immune system affect the neuropathology in PD. In PD patients [3, 4] and animal models [5–7], dopaminergic neuron loss is associated with increased numbers of activated microglia in the substantia nigra. These microglia produce proinflammatory mediators, such as interleukin-6 (IL-6), IL-1β, and nitric oxide that are suggestive of a chronic inflammatory state in PD [8–10]. In addition, PD patients exhibit aberrant adaptive immunity and increases in CD4+ and CD8+ T cells infiltrating the substantia nigra [3, 5, 11–16]. In addition, percentages of CD4+ T cells are diminished in PD patients relative to controls without disease [11–16], but frequencies of Th1 and Th17 effector T cells (Teffs) are increased within the CD4+ population [17]. These Teffs recognize nitrated-α-synuclein (N-α-Syn) as modified self-protein and exacerbate neuroinflammation and neurodegeneration [18, 19]. Furthermore, depletion of CD4+ T cells, but not CD8+ T cells, inhibits susceptibility to MPTP, neuroinflammation, and neuronal loss, thus underscoring the importance of CD4+ T cells in affecting progressive neurodegeneration [18, 20]. In addition to increased numbers of proinflammatory Teffs, frequencies of Th2 and regulatory T cells (Tregs) as well as Treg activity are diminished in PD patients compared to healthy subjects [13, 17]. Thus, for CD4+ T cells, opposite roles for CD4+ T cells include proinflammatory, neurotoxic processes and anti-inflammatory, neuroprotective functions; both of which can dictate the tempo of PD disease progression. Moreover, the translational implications of these findings are noteworthy as pro-inflammatory immune functions in PD patients can be controlled by CD4+ T cells for potential clinical benefit [21, 22].

Dendritic cells (DCs) are the antigen presenting cell (APC) responsible for induction of both Teffs and Tregs [23–27]. Granulocyte-macrophage colony-stimulating factor (GM-CSF) induces tolerogenic DCs from bone marrow cells, which in turn increase Treg number and function [28–32]. Furthermore, adoptive transfer of GM-CSF-induced tolerogenic bone marrow-derived DCs (BMDCs), diminish autoimmune responses and protect from development of autoimmune sequelae. In autoimmune disorders, Treg induction by tolerogenic BMDCs is mediated by OX40L/Jag-1-dependent mechanisms [33, 34]. In the 1-methyl-4-phenyl-1,2,3,6-tetrahydropyridine (MPTP) PD model, administration of GM-CSF increases numbers of Tregs without increasing the CD4+ T cell pool and attenuates neuroinflammation and neurodegeneration [35]. Additionally, Tregs from GM-CSF-treated mice are anti-inflammatory and neuroprotective in MPTP-intoxicated mice. In a recent phase 1 clinical trial, administration of sargramostim (recombinant human GM-CSF) to PD patients increased Treg number and activity, improved UPDRS, III scores, and normalized motor initiation signaling deficits [36]. Together, these data demonstrate that GM-CSF diminishes neuroinflammation and loss of dopaminergic neurons by increasing Treg numbers and function. As T cells express few, if any, receptors for GM-CSF, Treg induction by GM-CSF likely proceeds through indirect mechanisms. Moreover, those mechanisms must be achieved amid conditions of chronic exposure to GM-CSF, inflammation, and modified, misfolded proteins.

Our current study was designed to assess the effects of GM-CSF and modified α-synuclein (α-Syn) on tolerogenic DCs and Treg induction. Herein, we show that GM-CSF-induced tolerogenic BMDCs lead to induction of Tregs from CD4+ T cells pools. Stimulation of BMDCs with N-α-Syn upregulates expression of cytokine and chemokine genes and proteins, yet Treg induction capability, while diminished, remained at 88% of media controls. Further culture of BMDCs in GM-CSF significantly diminishes Treg induction to ≤55% of controls, regardless of stimulation with N-α-syn or intensity of proinflammatory mediator expression by BMDCs. In MPTP-intoxicated mice, adoptive transfer of tolerogenic BMDCs diminished numbers of reactive microglia and spared dopaminergic neurons along the nigrostriatal axis. While numbers of Tregs were not significantly increased after adoptive transfer of tolerogenic BMDCs to naïve mice, MPTP-intoxication after adoptive transfer significantly induced Treg transformation suggesting the necessity of acute inflammatory signaling to potentiate Treg induction. Together, the data provide a mechanism by which GM-CSF induces tolerogenic BMDCs that can drive T cell-to-Treg transformation, whereas further differentiation by continued exposure to GM-CSF and/or proinflammatory stimulation, diminish DC tolerogenic capacity. We posit that these processes provide a therapeutic strategy for BMDC-mediated control of Treg number and activity that lead to diminution of neuroinflammation and neurodegeneration in PD.

## Methods

### Animals

Male 6–8 week old mice C57BL/6J mice (Jackson Laboratories, Bar Harbor, ME) were used for all experiments. All procedures were performed in agreement with the National Institutes of Health guidelines and were approved by the University of Nebraska Medical Center Institutional Animal Care and Use Committee. Animals were maintained on a 12 h light/dark cycle and given food and water ad libitum.

### BMDC differentiation in vitro

Femurs from 6 to 8 week old male C57BL/6J mice were removed and washed two times in Hanks’ balanced salt solution (HBSS, Gibco, Waltham, MA) on ice and single cell suspensions of bone marrow cells (BMCs) were prepared by trituration. Red blood cells (RBCs) were lysed in ACK lysis buffer (Gibco) and washed in HBSS by centrifugation at 200 xg for 10 min. Cells were resuspended in R10 media [RPMI 1640 (Gibco) supplemented with 10% heat inactivated fetal bovine serum (Sigma, St. Louis, MO), 100 U penicillin, 100 μg/ml streptomycin (Gibco), 10 mM HEPES (Hyclone, Logan, UT), 2 mM L-glutamine (Gibco), and 55 nM 2-mercaptoethanol (Sigma)], and 4 × 10^6^ cells were cultured in 4 mls of R10 with 20 ng/ml of mouse recombinant GM-CSF (PeproTech, Rocky Hill, NJ) at 37 °C, 5% CO_2_ for 4 days. On days 4 and 6, used media was removed, the non-adherent cells harvested, and returned to culture with adherent cells in 4 mls R10 media supplemented with 20 ng/ml GM-CSF. On day 8, media was removed, cells washed and cultured in either R10 media alone or supplemented with 40 ng/ml recombinant mouse GM-CSF. On day 10, half of the BMDCs cultured in R10 media alone or R10 supplement with GM-CSF were stimulated with 30 μg/ml recombinant N-α-Syn [18] and cultured for 6 h for RNA isolation or 24 h for flow cytometric analysis of cells and supernatants for cytokine and chemokines. Thus, groups include GM-CSF-induced BMDCs further treated with 1) R10 media, 2) 20 ng/ml GM-CSF in R10 media, 3) R10 media followed by stimulation with 30 μg/ml N-α-Syn, and 4) 20 ng/ml GM-CSF in R10 media followed by stimulation with 30 μg/ml N-α-Syn. The timing of GM-CSF culture and N-α-Syn stimulation was chosen to affect maximal immune responses (data not shown).

### Isolation RNA, cDNA conversion and PCR arrays

Six hours after N-α-Syn stimulation, BMDCs were harvested, washed, and RNA isolated using the Rneasy mini kit (Qiagen, Germantown, MD) by the manufacturer’s protocol. RNA concentration was determined by UV spectroscopy at 260 nm and 280 nm (ND-100 Nanodrop spectrophotometer, Thermo Scientific, Waltham, MA). Five hundred nanograms of RNA was converted to cDNA using the RevertAID first strand cDNA synthesis kit (Thermo Scientific) following the manufacturer’s protocol. cDNA was added to molecular grade water (Invitrogen, Carlsbad, CA) and 2× RT^2^ SYBR green mastermix (Qiagen), and 25 μl of the mixture was added to each well of a Mouse Inflammatory Response and Autoimmunity array (PAMM-077ZA). PCR was performed in Eppendorf Realplex 2S Mastercycler starting at 95 °C for 10 min followed by 40 cycles of 95 °C for 15 s and 60 °C for 1 min. After 40 cycles, melting curve analysis was performed. The Ct values were determined and the ΔΔCt method was used to determine fold changes relative to cDNA derived from media cultured, unstimulated BMDCs samples using RT^2^ Profiler PCR array data analysis version 3.5.

### Flow cytometry

Twenty-four hours after culture with GM-CSF and/or N-α-Syn stimulation, BMDCs were detached by scraping, washed, and resuspended in 10 μg/ml rat gamma globulin in flow staining buffer (FSB) (0.5% bovine serum albumin (BSA) and 0.1% sodium azide in DPBS) for 40–60 min on ice to block Fc receptors. BMDCs were stained with the following mixture of AlexaFluor 488-anti-CD11c, PECy7-anti-CD11b, PE-anti-Jagged-1, APC-anti-OX40L, AlexaFluor 700-anti-MHC II, eFluor 450-anti-CD86, eFluor 710-anti-CD39 (eBioscience, San Diego, CA) and APC-Vio 770-anti-CD73 (Miltenyi Biotec, Auburn, CA) for 30 min at 4 °C. Cells were washed two times in FSB and were fixed with 1% formaldehyde in DPBS. Samples were analyzed with a BD LSR II flow cytometer and FACSDiva software (BD biosciences, San Jose, CA) at the University of Nebraska Medical Center Flow Cytometry Research Facility. From the single cell-gated population, the percentages of positive for CD11c and CD11b were determined by drawing a gate that comprised 98% of the isotype control as negative. The geometric mean fluorescent intensity (MFI) of each surface marker was determined for CD11c+ cells.

### Luminex array

After 24 h of culture with GM-CSF and/or N-α-Syn stimulation of BMDCs, the supernatant was removed and clarified by centrifugation at 10,000 xg for 5 min. Cytokine and chemokine concentrations were determined by Luminex xMAP Mouse cytokine and chemokine magnetic bead kit (Millipore, Billerica, MA) according to the manufacturer’s protocol. Briefly, 25 μl of supernatant from each of the 7 replicates for all treatment groups was added to a 96-well plate in duplicate or triplicate. To each sample or standard was added 25 μl containing antibodies to IFNγ, IL-1α, IL-1β, IL-2, IL-4, IL-5, IL-6, IL-7, IL-9, IL-10, IL-12p40, IL-12p70, IL-13, Lix, IL-15, IL-17, IP-10, MIP-2, MIG, RANTES, and TNF-α and incubated at 4 °C overnight. The plates were washed two times, detection antibodies added, incubated at room temperature (RT) for 60 min, streptavidin-PE was added, and incubated at RT for 30 min. The plate was washed two more times and analyzed with the Millipore Magpix system with Luminex Xponent 4.2 software. The concentration for each protein was determined from a standard curve.

### Isolation of CD4+ cells and co-culture

For BMDC and CD4+ T cell co-culture, GM-CSF-induced BMDCs were harvested after culture in R10 media alone or R10 media supplemented with GM-CSF, unstimulated, or stimulated with 30 μg/ml N-α-Syn. CD4+ cells (93% CD4+CD25-Foxp3- and 2%  CD4+CD25^hi^Foxp3+) were isolated from spleens of C57BL/6J mice using the Miltenyi CD4+ negative selection kit (Miltenyi Biotech). Into the wells of a 24-well plate, 2 × 10^6^ CD4+ cells and 1 × 10^6^ BMDCs were combined and incubated at 37 °C with 5% CO_2_ for 5 days. Non-adherent cells were removed and stained with PECy7-anti-CD4 and PE-anti-CD25 (eBioscience) and cells were permeabilized and fixed using the eBioscience Foxp3/transcription factor staining buffer set according to the manufacturer’s protocol for 1 h prior to staining with APC-anti-Foxp3(eBioscience). Cells were analyzed with a LSR II flow cytometer (BD). Using FACSDIVA software (BD), single cells were gated to include CD4+ T cells, quadrants were set to include 98% of the isotype control, and the percentages of CD25^hi^Foxp3+ Tregs were determined.

### Treg functional assay

The ability of Tregs to suppress the proliferation of CFSE-labelled CD4+CD25- T responder cells (Tresps) was performed as described previously [13, 37]. Briefly, CD4+CD25- and CD4+CD25+ T cells were isolated using the Treg isolation kit (Miltenyi) from mouse spleens and from non-adherent cells after 5 days of co-culture of media-cultured, unstimulated BMDCs and CD4+ T cells. Splenic CD4+CD25- Tresps were labelled with CFSE (CellTrace Cell Proliferation Kit, Thermo Fisher, Waltham, MA) according to the manufacturer’s protocol. In a 96-well U bottom plate, 50,000 CD4+CD25+ Tregs were diluted by serial 2-fold dilutions. To each well was added 50,000 CFSE-labelled CD4+CD25- Tresps, to yield Tresp:Treg ratios of 1:1, 1:0.5, 1:0.25, and 1:0.125. Each well received 50,000 Dynabeads mouse transactivator CD3/CD28 beads (Gibco) and the 96-well plate was incubated at 37 °C, 5% CO_2_ for 3 days. The cells were fixed by removing half the media and adding in 1% formaldehyde in DPBS prior to flow cytometric analysis using a LSR II flow cytometer and FACSDIVA software (BD).

### MPTP and adoptive transfer

Media-cultured, unstimulated tolerogenic BMDCs were prepared by culture of BMCs in 20 ng/ml GM-CSF for 8 days followed by 3 days of culture in R10 media alone. To male C57BL/6J mice, 1.5 × 10^6^ BMDCs in 250 μl of DPBS was injected intravenously (i.v.) into the tail vein at two and one week prior to MPTP intoxication. Mice were treated with 4 doses of either 10 ml/kg DPBS (Gibco) or 16 mg free base MPTP (MPTP-HCl, Sigma-Aldrich, St. Louis, MO)/kg with each dose administered subcutaneously (s.c.) every 2 h. MPTP safety and handling protocols were followed [38].

### Perfusion and immunohistochemistry for Mac-1 and tyrosine hydroxylase (TH)

Mice were sacrificed 2 days post MPTP intoxication to assess neuroinflammation and 7 days post MPTP to assess neuronal survival [35, 37]. Briefly, mice were terminally anesthetized with pentobarbital (Vortech, Dearborn, MI), transcardially perfused with DPBS, and fixed with 4% paraformaldehyde/DPBS. Brains were removed, post-fixed for 24 h in 4% paraformaldehyde, cryoprotected for 2 days in 30% sucrose/DPBS, and snap-frozen. Tissues were cryosectioned at 30 μm sections through the striatum and midbrain containing the substantia nigra and processed for immunohistochemistry [35, 37]. Free-floating sections were blocked of endogenous peroxidases with 3% hydrogen peroxide and non-specific activity in 5% normal goat serum (Vector Laboratories, Burlingame, CA). Blocked sections were reacted with anti-TH (EMD/Milipore, Burlington, MA) at 1:2000 dilution for striatal sections and 1:1000 dilution for substantia nigra sections. After 48 h, sections were washed and reacted with 1:400 dilution of goat-anti-rabbit secondary antibody (Vector Laboratories) followed by ABC biotin-avidin peroxidase solution (Vector Laboratories) prior to color generation with 3,3′-diaminobenzidine (DAB, Sigma). Sections containing substantia nigra were counter stained for Nissl substance. For Mac-1 staining, sections were incubated with a 1:500 dilution of anti-Mac-1 primary antibody (Bio-Rad, Hercules, CA) overnight. Sections were washed and reacted with 1:500 dilution of biotinylated rabbit anti-rat antibody (Vector laboratories) followed ABC biotin-avidin peroxidase solution (Vector Laboratories) prior to color generation with DAB. Numbers of dopaminergic neurons (TH+Nissl+), non-dopaminergic neurons (TH-Nissl+), and reactive microglia (amoeboid Mac-1+) were estimated by stereological analyses analysis using the optical fractionator module of StereoInvestigator (MBF Bioscience, Williston, VT) [35, 37]. Densities of TH+ striatal termini were determined using digital densitometry using ImageJ as described [35, 37].

### Isolation of RNA from midbrain for PCR array

Two days after MPTP intoxication, mice were sacrificed, brains quickly removed, hemisected, midbrain dissected, placed in RNAlater (Thermo Fisher), tissues weighed, and flash frozen at − 80 °C. To isolate RNA, midbrains were homogenized in 350 μl β-mercaptoethanol-supplemented RLT buffer (Qiagen) for every 30 mg tissue. Tissues were sequentially drawn up and down through 18, 20 and 27 Ga needles. RNA was isolated, converted to cDNA and assessed using Mouse Inflammatory Response and Autoimmunity PCR arrays as described.

### Statistics

Statistics were performed using Prism GraphPad version 6. Means and SEM were determined for release of cytokines from BMDCs, relative changes in mean fluorescent intensity (MFI) in the flow markers on BMDCs, Treg frequency in the CD4+ population, and Mac-1+ microglia. For TH+ neuron counts and striatal densities, means and SEM were determined for values within the 99% confidence interval. For all analyses, one-way ANOVAs were performed followed by the appropriate post-hoc test adjusted for multiple comparisons. A *p* value less than or equal to 0.05 was selected as significant.

## Results

### GM-CSF and BMDCs

**Fig. 1:**
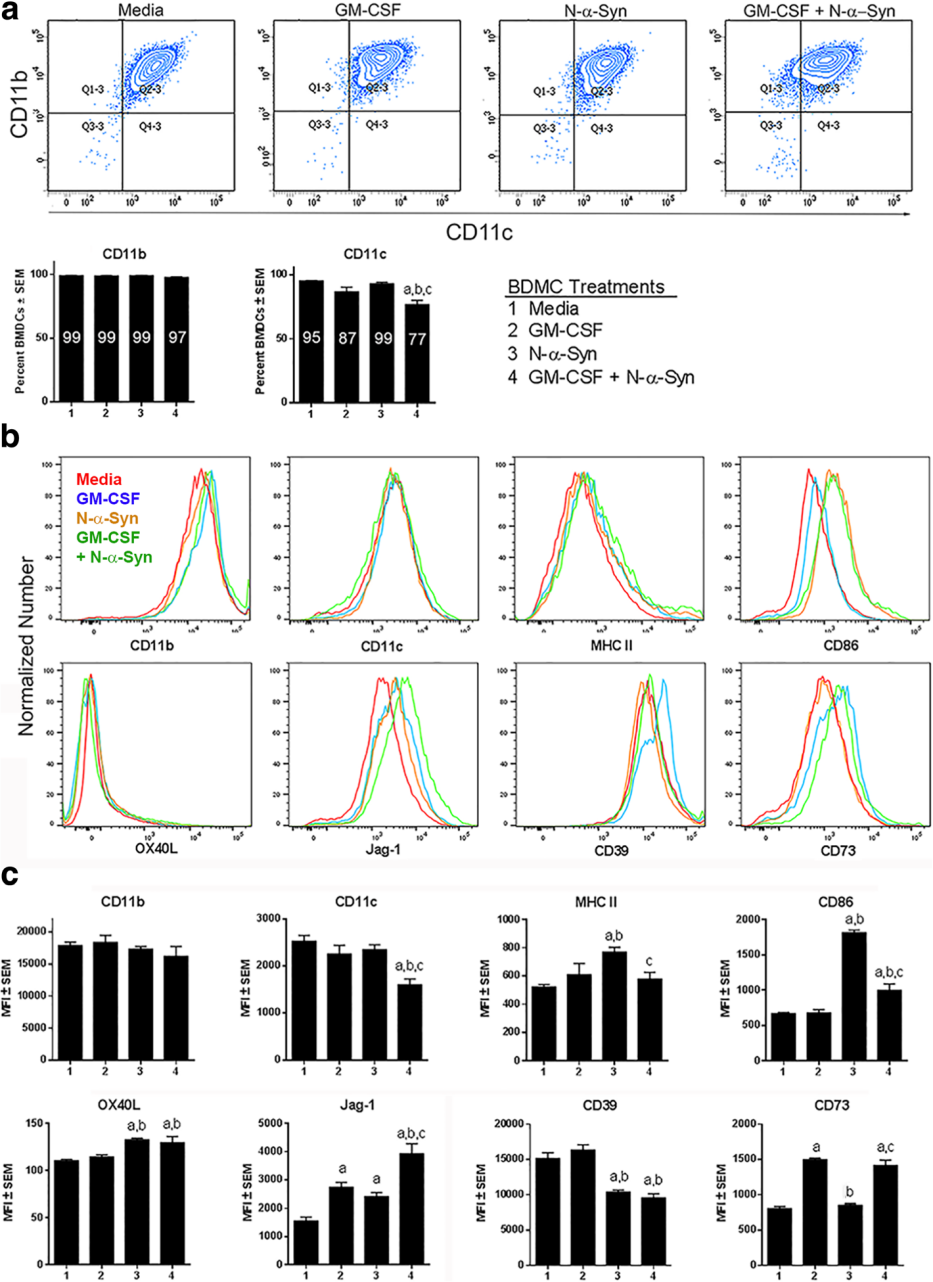
Surface expression on BMDCs**.** GM-CSF-generated BMDCs were cultured in media alone or with 20 ng/ml GM-CSF for 2 days prior to stimulation with 30 μg/ml N-α-Syn for 1 day. Treatment groups were as follows: (1) media-cultured, unstimulated BMDCs; (2) GM-CSF-cultured, unstimulated BMDCs; (3) media-cultured, N-α-Syn-stimulated BMDCs; and (4) GM-CSF-cultured, N-α-Syn-stimulated BMDCs. Cells were harvested and reacted with antibodies to detect expression of CD11c, CD11b, MHC II, CD86, OX40L, Jag-1, CD39 and CD73, then evaluated by flow cytometric analysis. **a** Cells were gated by forward scatter area vs height to include only single cells and  CD11b+CD11c+ BMDC populations were identified. Percentages of cells expressing CD11b or CD11c were determined and the mean percentages of single cells positive for each marker are shown within the bars. (**b** and **c**) BMDCs were gated to include the CD11b+CD11c+ cell population and the geometric mean fluorescent intensity (MFI) was determined for expression of MHC II, CD86, OX40L, Jag-1, CD39, and CD73. **b** Overlays of representative histograms are shown for BMDCs treated with media (red), GM-CSF (blue), N-α-Syn (orange), or GM-CSF + N-α-Syn (green). **c** Histograms represent the means ± SEM for 7 replicates from each treatment group. The means were compared by one-way ANOVA and Newman-Keuls post-hoc test whereby *p* ≤ .0.05 compared to BMDCs treated with ^a^media, ^b^GM-CSF, or ^c^N-α-Syn

Because tolerogenic DCs exhibit decreased expression of proinflammatory cytokines in response to maturation stimuli [39, 40], we tested the expression of co-stimulatory molecules to determine whether GM-CSF mitigates these responses. Bone marrow cells were cultured for 8 days in 20 ng/ml GM-CSF to produce immature bone marrow-derived dendritic cells (BMDCs). Immature BMDCs were ≥ 95% CD11b+CD11c+ (data not shown). To assess cellular phenotypic changes beyond the initial 8 days of culture, immature BMDCs were maintained in media alone or supplemented with GM-CSF for 2 days and/or stimulated with N-α-Syn for 1 day. Frequencies and fluorescent intensities of cell surface markers were assessed by flow cytometric analysis. Frequencies of cells that express the dendritic cell markers CD11b and CD11c showed little change, if any, regardless of culture conditions (Fig. 1a). CD11b expression was retained by ≥97% of the BMDCs regardless of treatment. Greater than 85% of immature BMDCs expressed CD11c after culture in media, GM-CSF, or stimulation with N-α-Syn, while culture in GM-CSF and N-α-Syn stimulation reduced the number of CD11c+ BMDCs to 77% (Fig. 1a). This was confirmed by loss in the MFI of BMDCs expressing CD11c (Fig. 1b and c). Overall, virtually all BMDCs were myeloid DCs that stably expressed CD11b and CD11c independent of culture conditions.

We next tested whether continued GM-CSF exposure and/or stimulation with N-α-Syn induced phenotypic changes of co-stimulatory and regulatory molecules by evaluating mean fluorescent intensity (MFI) compared to media treatment (Fig. 1b and c). Notably, fluorescent intensities associated with cell surface expression were normally distributed as shown for representative histograms (Fig. 1b). Gating on CD11b+CD11c+ BMDCs, the geometric MFIs of MHC II and CD86 expression were determined. Expression of MHC II was significantly elevated after N-α-Syn stimulation compared to culture in media alone or GM-CSF (Fig. 1c). Interestingly, continued culture with GM-CSF prior to N-α-Syn stimulation significantly diminished MHC II expression compared to stimulation with N-α-Syn alone. Compared to culture in media or GM-CSF alone, MFI of CD86 was significantly elevated by BMDCs stimulated with N-α-Syn or treated with GM-CSF and N-α-Syn combined, which was significantly diminished from BMDCs stimulated with N-α-Syn alone.

The expression of co-stimulatory molecules associated with anti-inflammatory or regulatory activities were also tested as those molecules could skew naïve CD4+ T cells towards Th2 and Treg transformation. The surface expression of OX40L and Jag-1 has been linked to Treg induction [33, 34]. OX40L expression was slightly, though significantly altered by treatment with N-α-syn with or without prior exposure to GM-CSF (Fig. 1b and c). Compared to media control, Jag-1 expression was significantly increased by all other culture conditions. Stimulation with N-α-Syn increased expression by 1.5-fold, and continued cultivation with GM-CSF by 1.8-fold, while treatment with both increased expression by 2.5-fold. Together, these data show that OX40L expression showed limited change by any culture condition; however, Jag-1 expression was increased by treatment with GM-CSF and/or N-α-Syn.

Lastly, we tested the expression of surface ATPases since metabolism of extracellular ATP to adenosine is associated with the generation of Tregs [41]. CD39 is an ectonuclease which metabolizes ATP to AMP and CD73 is a surface expressed enzyme which metabolizes AMP to adenosine. Stimulation with N-α-syn with or without further GM-CSF exposure diminished the expression of CD39 by 37 and 32%, respectively compared to treatment with media alone (Fig. 1b and c). In contrast, further exposure to GM-CSF regardless of stimulation with N-α-Syn increased CD73 expression by at least 1.8-fold compared to media controls, whereas stimulation with N-α-Syn alone yielded no significant increase in CD73 expression. This suggested that stimulation with N-α-Syn reduces some regulatory components of Treg formation, whereas GM-CSF may increase others. Together, these results support the notion that GM-CSF affects the BMDC tolerogenic state defined by diminished surface expression of MHC II and CD86 as well as increased Jag-1 and CD73 expression and that N-α-Syn stimulation and GM-CSF may increase or decrease co-stimulatory or regulatory components necessary for Treg induction.

### N-α-Syn stimulated BMDCs with or without culture in GM-CSF potentiates an inflammatory state

**Fig. 2:**
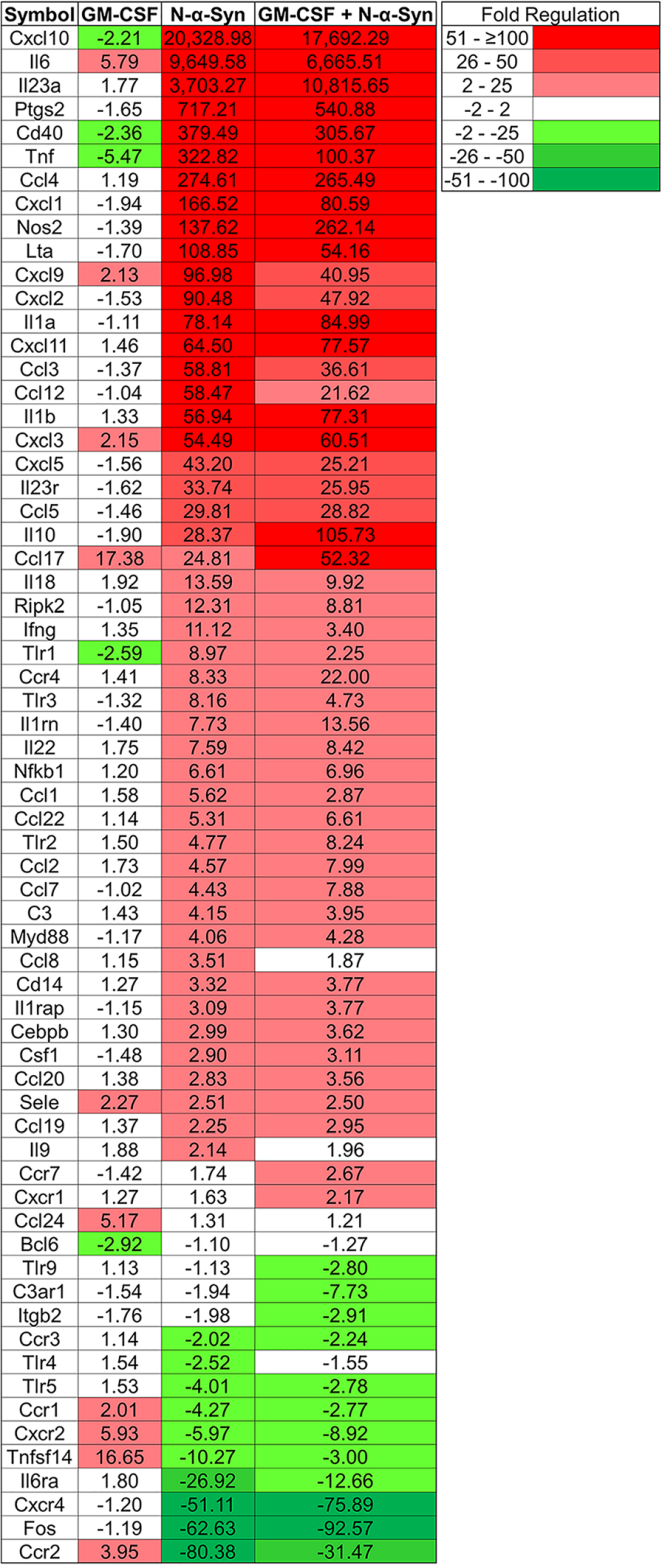
Gene expression of GM-CSF, N-α-Syn, or GM-CSF/N-α-Syn-cultured BMDCs**.** GM-CSF-generated BMDCs were cultured for 2 days in media or 20 ng/ml GM-CSF (**GM-CSF**). BMDC cultures from each group were stimulated with 30 μg/ml N-α-Syn for 6 h (**N-α-Syn** or **GM-CSF + N-α-Syn**, respectively). RNA was isolated, converted to cDNA and assessed by PCR arrays for proinflammatory genes. Gene expression from BMDCs from treatment groups was determined relative to BMDCs treated with media alone. Each treatment group was composed of 6 replicates and fold change was determined by SA Bioscience array software

As N-α-Syn stimulation increases BMDC co-stimulatory molecule expression, we tested the effects of GM-CSF, N-α-Syn, or both on cellular RNA immune profiles. cDNA from RNA was analyzed using a PCR array for regulation of proinflammatory cytokine and chemokine genes, and normalized to BMDCs cultured in media alone. Continuing culture of BMDCs in GM-CSF increased the expression by more than two-fold for *Il6*, *Cxcl9*, *Cxcl3*, *Ccl17*, *Sele, Ccl24, Ccr1, Cxcr2*, *Tnfsf14,* and *Ccr2*, but down-regulated by more than two-fold *Cxcl10, Cd40, Tnf, Tlr1,* and *Bcl6* (Fig. 2). Stimulation of BMDCs with N-α-Syn with or without GM-CSF increased expression of maturation markers *Ccr7* and *Cebpb* confirming that N-α-Syn alone activated BMDCs. Also, N-α-Syn stimulation increased expression of the proinflammatory genes *Cxcl10, Il6, Il23a, Tnf, Ccl4, Cxcl1, Cxcl2*, *Il1a, Il1b, Il18,* and *Ifng*. Several genes involved in Toll-like receptor (TLR) signaling including *Tlr1, Tlr3, Tlr2, Cd14, Myd88,* and *Nfkb1* were increased following N-α-Syn stimulation while *Tlr4* and *Tlr5* were decreased. Moreover, N-α-Syn stimulation also increases *Nos2* expression, the gene for inducible nitric oxide synthase. In contrast, several down-regulated genes by N-α-Syn stimulation included *Ccr2, Fos, Cxcr4, Il6ra*, *Tnfsf14, Cxcr2,* and *Ccr1*.

Compared to N-α-Syn stimulation of BMDCs, continued culture with GM-CSF prior to N-α-Syn treatment decreased expression of *Cxcl10, Il6, Ptgs2, Tnf, Cxcl1,* and *Lta* (Fig. 2). Conversely, genes whose expression increased were *Il23a, Nos2, Il1a*, *Cxcl11, Il1b, Cxcl3*, *Il10,* and *Ccl17*. *Cxcr4* and *Fos* were modestly down-regulated. Prolonged culture with GM-CSF prior to N-α-Syn stimulation, increased expression of *Tlr2* and decreased the expression of *Tlr1, Tlr3*, however *Myd88*, *Cd14*, and *Nfkb1* were not changed. These changes demonstrate that continued culture with GM-CSF did not diminish the ability of BMDCs to respond to N-α-Syn stimulation, but rather altered the expression of select proinflammatory genes and may target specific genes expressed after stimulation.

**Fig. 3:**
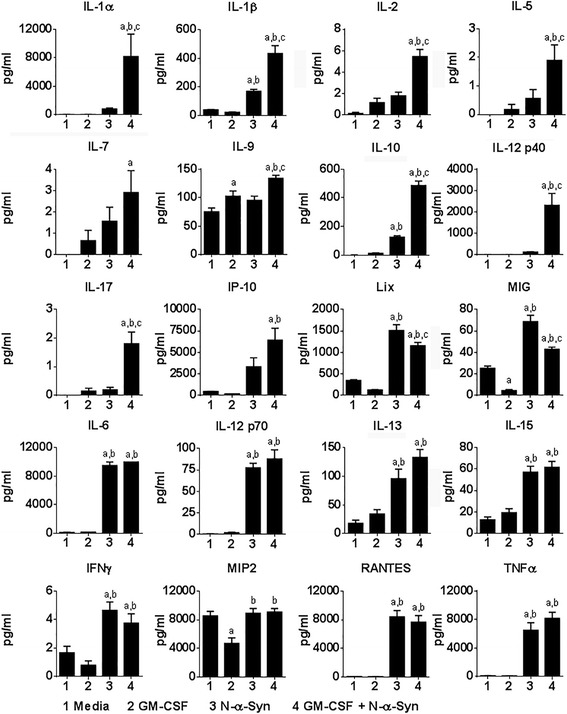
Cytokines and chemokines released from BMDCs. GM-CSF-generated BMDCs were pretreated with media or 20 ng/ml GM-CSF for 2 days and/or stimulated with 30 μg/ml N-α-Syn for 1 day. Treatment groups were as follows: (1) media-cultured, unstimulated BMDCs; (2) GM-CSF-cultured, unstimulated BMDCs; (3) media-cultured, N-α-Syn-stimulated BMDCs; and (4) GM-CSF-cultured, N-α-Syn-stimulated BMDCs. Supernatants were removed and assessed by Luminex xMAP proinflammatory cytokine and chemokine array. For each treatment condition,19–21 replicates were included in the array analyses. Means ± SEM of protein concentrations were assessed by one-way ANOVA and Tukey’s post-hoc test whereby *p* ≤ .0.05 compared to BMDCs treated with ^a^media, ^b^GM-CSF, or ^c^N-α-Syn

To further test prolonged GM-CSF exposure and N-α-Syn stimulation on BMDCs, we assessed cytokine and chemokine production from culture supernatants. Continued culture in GM-CSF produced few significant changes in cytokine or chemokine production with the exception of diminished MIP2 and increased IL-9 (Fig. 3). N-α-Syn stimulation in media alone significantly increased release of IL-1β, IL-10, Lix, MIG, IL-6, IL-12p70, IL-13, IL-15, IFN-γ, RANTES, and TNFα. Continued culture with GM-CSF prior to N-α-Syn increased release of IL-1α, IL-1β, IL-2, IL-5, IL-7, IL-9, IL-10, IL-12p40, IL-17, and IP-10, and decreased release of Lix and MIG compared to media control and N-α-Syn stimulation. Culture of BMDCs with GM-CSF prior to N-α-Syn stimulation did not affect production or release  of pro-inflammatory cytokines. In addition, IL-10 was increased by continued GM-CSF culture. Together, these data suggest that after induction of BMDCs by GM-CSF, further culture with or without GM-CSF does not change the cytokine and chemokine levels. However, stimulation of BMDCs with N-α-Syn, regardless of culture conditions elicits a proinflammatory profile. Moreover, whether N-α-Syn affects Treg induction by GM-CSF-induced tolerogenic DCs remains unanswered.

### BMDC-mediated induction of Tregs diminishes with continued presence of GM-CSF

**Fig. 4:**
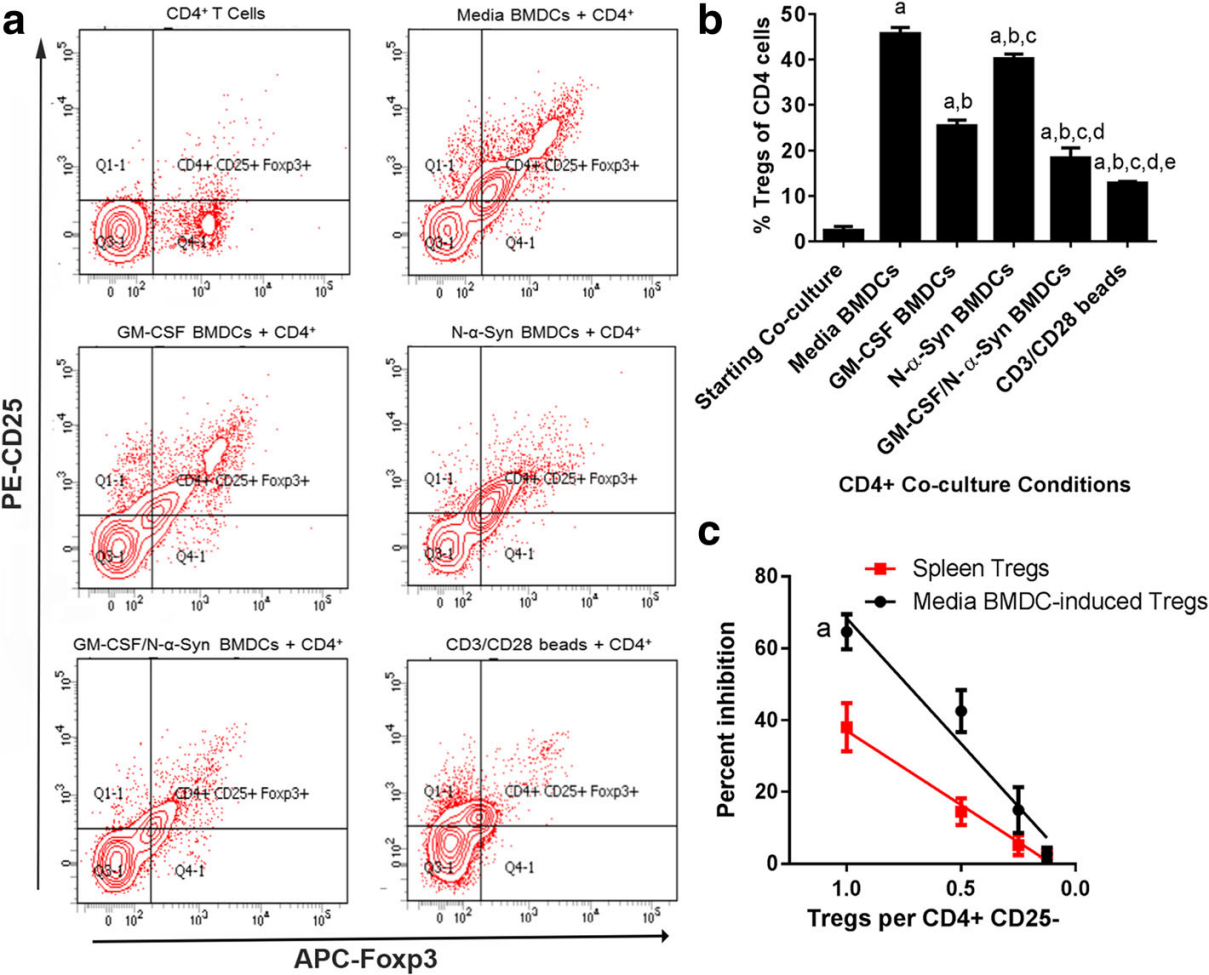
BMDCs induce functional Tregs. GM-CSF-generated BMDCs were pretreated with media or 20 ng/ml GM-CSF for 2 days and/or stimulated with 30 μg/ml N-α-Syn for 1 day prior to co-culture for 5 days with CD4+ T cells. Non-adherent T cells were harvested, stained for expression of CD4, CD25, and Foxp3, and evaluated by flow cytometric analysis. CD4+ gate was drawn to include > 98% of the populations and quadrants were placed to include 98% of the isotype control. **a** Contour plots of flow cytometric analyses for expression of Foxp3 and CD25 by CD4+ T cells from 5 day co-cultures of BMDCs and CD4+CD25- T cells. **b** Quantitation of CD25+Foxp3+ Tregs within CD4+ T cell population from BMDC-CD4+ T cell co-cultures or treated with CD3/CD28 transactivation beads. Mean percentages ± SEM of Tregs were determined for 3 replicate co-cultures and evaluated by one-way ANOVA followed by Newman-Keul’s post-hoc test whereby *p* ≤ 0.05 compared to ^a^starting CD4+ T cells; ^b^media-cultured BMDCs/CD4+ T cells; ^c^GM-CSF-cultured BMDCs/CD4+ T cells; ^d^media-cultured, N-α-Syn-stimulated BMDCs/CD4+ T cells; and ^e^GM-CSF cultured, N-α-syn stimulated BMDCs/CD4+ T cells. **c** Tregs were enriched by magnetic bead selection for CD4+CD25+ T cells either from 5 day media co-cultures of BMDCs/CD4+ T cells or from primary isolates of splenic T cells from naïve mice. Tregs were diluted by serial 2-fold dilutions, added to CFSE-labelled CD4+CD25- cells, and stimulated with CD3/CD28 tansactivator beads for 3 days. Cells were harvested and assessed by flow cytometric analysis for Treg activity to suppress T cell proliferation. Linear regression showed that Treg activity correlated with Treg dose for BMDC-induced Tregs (r^2^ = 0.7941, F = 80.97, DFn = 1, DFd = 21, *p* < 0.00001) and for primary splenic Tregs (r^2^ = 0.6815, F = 47.07, DFn = 1, DFd = 22, *p* < 0.0001). Additionally, comparison of the two regression analyses show that the lines are significantly different (F = 8.52, DFn = 1, DFd = 43, *p* = 0.0056)

To determine culture conditions necessary for Treg induction by tolerogenic DCs and to test these processes, GM-CSF-induced BMDCs were co-cultured with CD4+ T cells from naïve mice for 5 days. After co-culture, non-adherent cells were analyzed by flow cytometric analysis to determine frequencies of Tregs (CD4+CD25+Foxp3+). Co-culture of T cells with BMDCs regardless of conditions increased Treg frequencies within the CD4+ T cell population compared to within the initiating CD4+ isolates (Fig. 4a and b). Notably, T cells co-cultured with BMDCs in media alone generated a greater frequency of Tregs than any culture condition. Co-culture in the presence of N-α-Syn induced levels of Tregs similar though significantly less than those of media controls. Co-culture of BMDCs and T cells in GM-CSF or GM-CSF and N-α-Syn combined produced Treg frequencies less than 55% of those co-cultured in media alone (Fig. 4a and b). To test the role of classical T cell activation and signaling pathways, CD4+ T cells from naïve mice were stimulated with CD3/CD28 transactivator beads. While Treg frequencies were significantly increased relative to initiating CD4+ T cell isolates, the levels were significantly less than any other treatment. Together, these results demonstrate that BMDCs can induce Tregs from CD4+ T cells. The presence of N-α-Syn during induction slightly diminishes Treg production despite increased levels of proinflammatory cytokines. However, co-culture in the presence of GM-CSF, with or without N-α-Syn, significantly diminishes Treg induction by 55 or 46%, respectively, despite little, if any proinflammatory cytokines produced by culture in GM-CSF alone. The data also suggest that processes for BMDC-mediated induction of Tregs require variant signals than those generated by CD3/CD28-mediated activation and proliferation of CD4+ T cells.

We next tested whether Tregs produced by BMDCs were functional. Non-adherent T cells from BMDC co-cultures were enriched for CD4+CD25+ T cells, serially-diluted, co-cultured with CFSE-stained CD4+CD25- Tresps, stimulated with CD3/CD28 transactivator beads, and T cell proliferation assessed. Also, fresh isolates of CD4+CD25+ Tregs were enriched from spleens of naïve mice, and similarly assessed to compare functional capacities of Treg populations. Tregs from either source were capable of suppressing proliferation of Tresps in a dose dependent fashion (Fig. 4c). Notably, Tregs from BMDC co-cultures showed greater suppressive ability than primary Treg isolates. Together, these results demonstrated that BMDCs produce functional CD4+CD25+Foxp3+ Tregs.

### BMDCs are neuroprotective

**Fig. 5:**
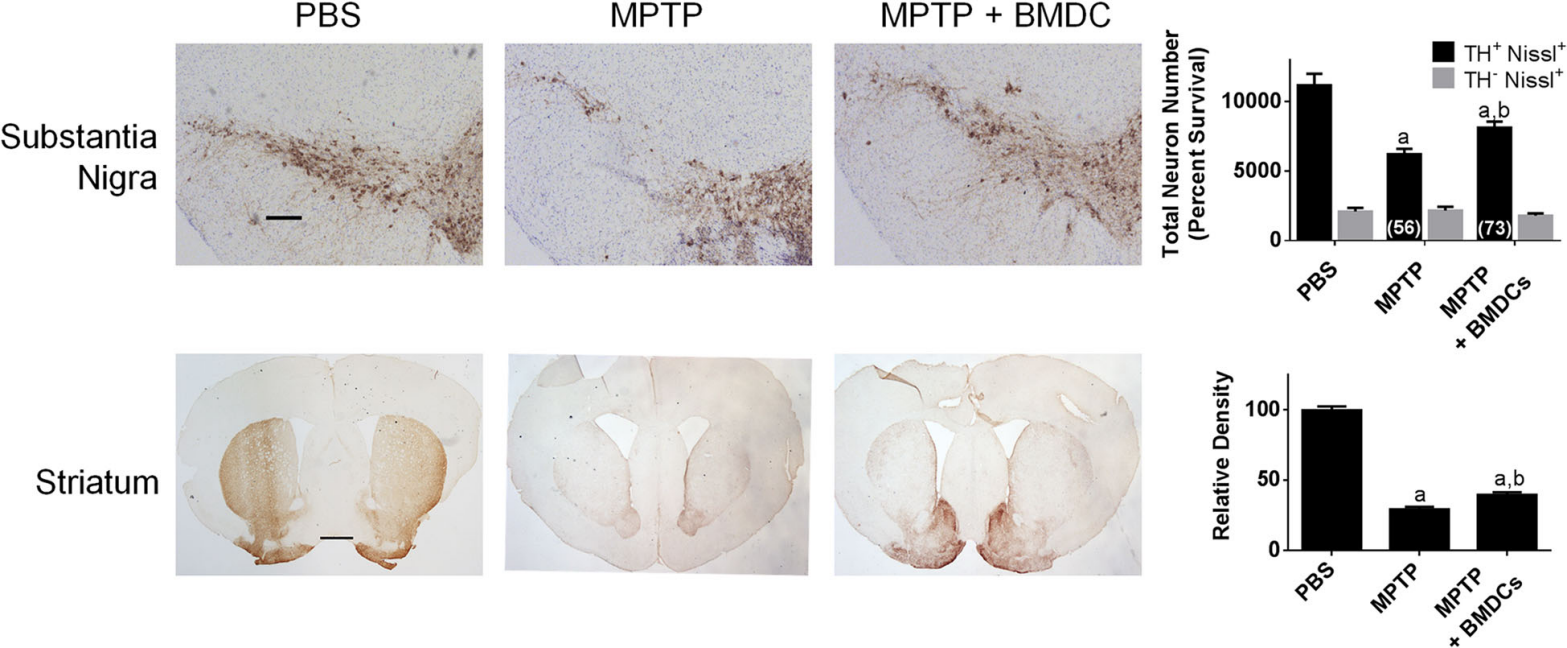
BMDCs are neuroprotective in the MPTP mouse model. GM-CSF-generated BMDCs were cultured in media alone, harvested, and 1.5 × 10^6^ in 0.25 mls were transferred i.v. at one and two weeks prior to intoxication with four 16 mg/kg doses of MPTP. Mice that received PBS or MPTP alone served as controls. Seven days after MPTP intoxication, brains were removed, frozen, and cryosectioned at 30 μm/section to contain the substantia nigra and striatum. Sections were stained by immunohistochemistry for tyrosine hydroxylase (TH) and nigral sections were counter stained for Nissl substance. Scale bar is 200 μm. Total dopaminergic neurons (TH+Nissl+) and non-dopaminergic neurons (TH-Nissl+) in the substantia nigra were estimated by stereological analysis for mice treated with PBS (*n* = 6), MPTP (*n* = 8), or BMDCs and MPTP (*n* = 7). Scale bar is 1 mm. Density of TH termini in the striatum was determined for 1.4 mm^2^ area for 4–6 sections for each striatum from mice treated with PBS (n = 7), MPTP (n = 7), or BMDCs and MPTP (n = 6). Relative densities were normalized to that of the PBS control (100% relative density). Means ± SEM of total number of nigral neurons or means ± SEM) of relative striatal TH densities were assessed by one-way ANOVA followed by Tukey’s post hoc test whereby *p* ≤ 0.05 compared to mice treated ^a^PBS or ^b^MPTP alone

Prior studies demonstrated that Tregs are neuroprotective [35, 42–45]. Moreover, tolerogenic BMDCs induce Tregs in models of autoimmunity and neurodegeneration [28–32]. Thus, we tested whether BMDCs could protect dopaminergic neurons from MPTP intoxication. GM-CSF-differentiated BMDCs were cultured in media alone and adoptively transferred into mice one and two weeks prior to MPTP intoxication. Results were compared to those from mice treated with MPTP or PBS alone. Seven days after MPTP intoxication and resolution of cellular debris [46], numbers of surviving nigral TH+ dopaminergic neuronal bodies and striatal termini were evaluated. Compared to MPTP-treated animals, increased numbers of TH+ neurons in the substantia nigra and densities of striatal termini survived from animals in which BMDCs were adoptively transferred (Fig. 5).

**Fig. 6:**
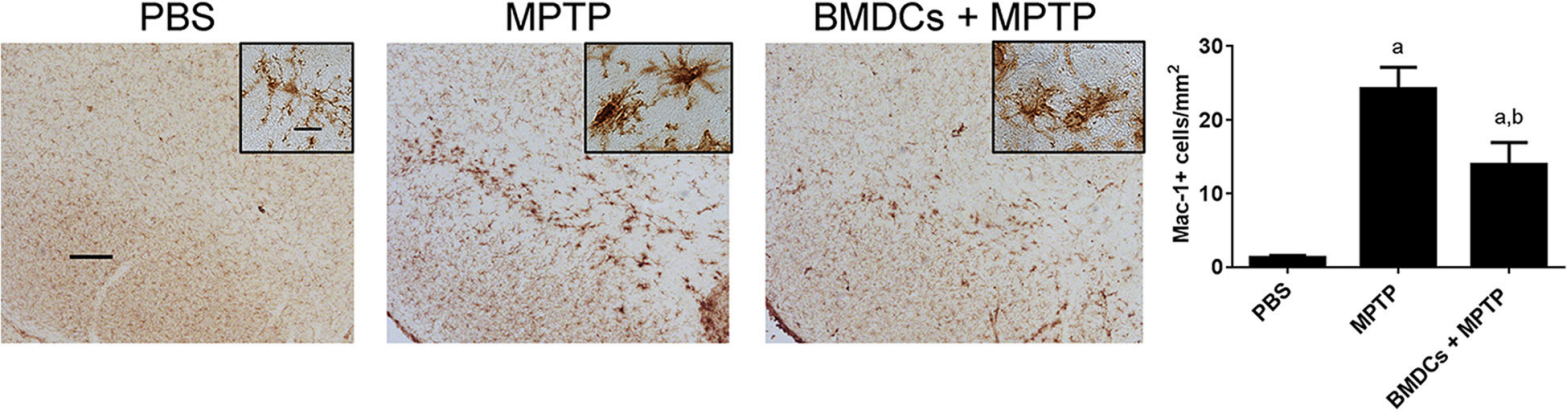
BMDCs decrease the number of reactive microglia in the MPTP model**.** GM-CSF-generated BMDCs were cultured for 3 days in media alone, harvested, and 1.5 × 10^6^ in 0.25 mls were transferred i.v. at one and two weeks prior to intoxication with four 16 mg/kg doses of MPTP. Mice treated with PBS or MPTP alone served as controls. Two days after MPTP intoxication, mice were sacrificed, brains removed, frozen, and cryosectioned at 30 μm/section through the midbrain containing the substantia nigra. Sections were stained for Mac-1 expression by microglia. Scale bar for the larger image is 200 μm and the scale bar in the inset is 20 μm. Densities of reactive microglia (ameboid morphology and increased expression of Mac-1) per area were estimated from 4 to 6 sections/animal by stereological analysis of the substantia nigra for mice treated with PBS (*n* = 5), MPTP (n = 5), or BMDCs and MPTP (n = 5). Mean densities ± SEM were compared by one-way ANOVA followed by Tukey’s post hoc test whereby *p* ≤ 0.05 compared to mice treated with ^a^PBS or ^b^MPTP

As diminished numbers of reactive microglia correlate with dopaminergic neuroprotection [6, 13, 35, 37, 47, 48], we assessed the abilities of GM-CSF-generated BMDCs to affect neuroinflammation. Mice treated with PBS, MPTP, or BMDCs prior to MPTP were compared 3 days after MPTP intoxication; the time of peak neuroinflammation [46, 49]. Adoptive transfer of BMDCs significantly decreased numbers of reactive microglia by 58% compared to MPTP-treated controls (Fig. 6). These data support the capacity of BMDCs to diminish neuroinflammatory processes during MPTP-induced neurodegeneration.

**Fig. 7:**
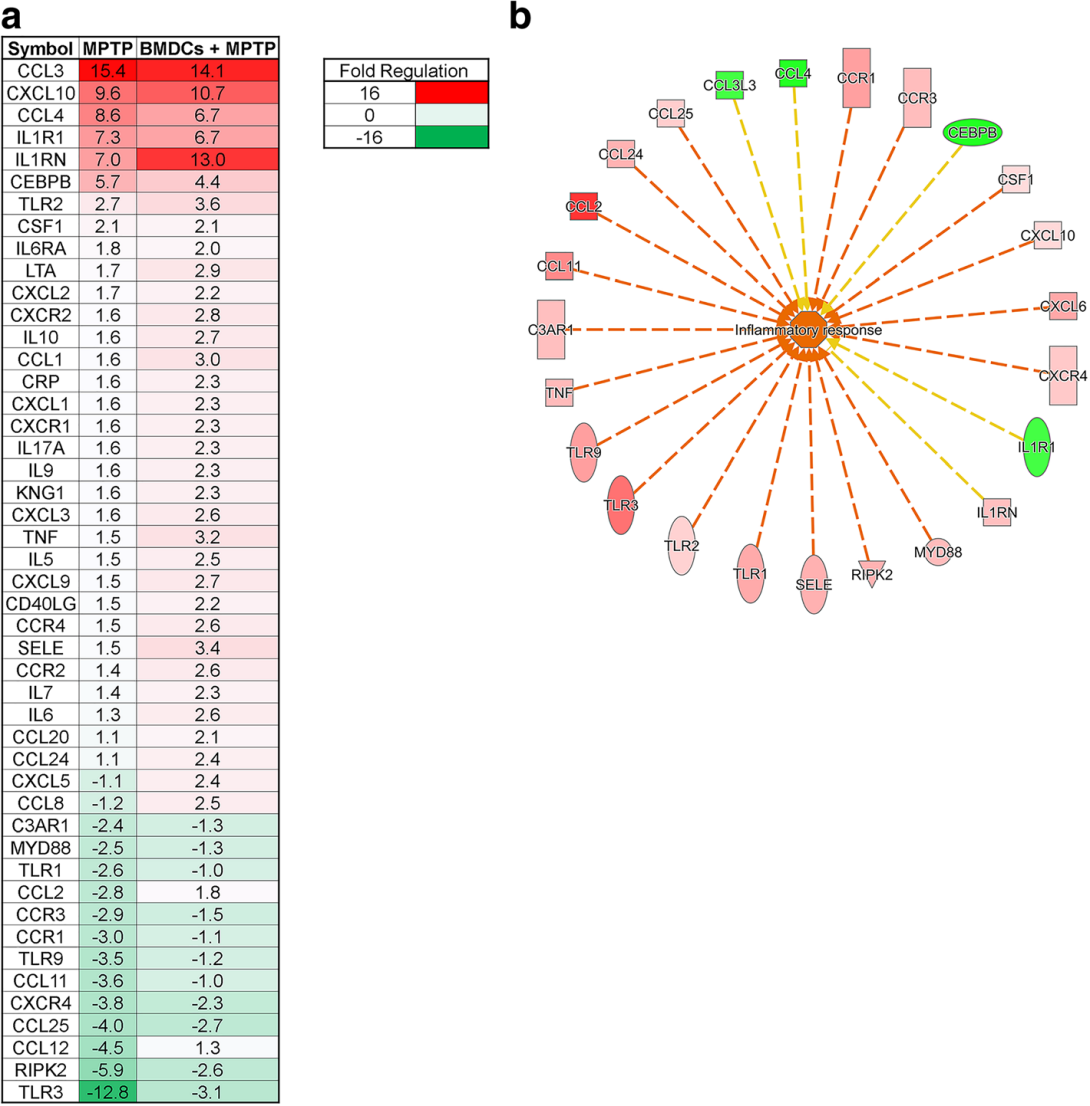
Gene expression in midbrain after treatment with MPTP or BMDCs and MPTP. BMDCs were differentiated for 8 days in 20 ng/ml GM-CSF prior to 3 days of pretreatment in media alone. These BMDCs were transferred i.v. one and two weeks prior to intoxication with four doses of 16 mg/kg MPTP. Two days after intoxication, PBS, MPTP and BMDC + MPTP mice were sacrificed and the brain was removed, hemisected and the midbrain was incubated in RNAlater for 24 h prior to freezing at − 80 °C. RNA was isolated from the midbrain, converted to cDNA PCR arrays of proinflammatory genes were run. **a** Gene expression was determined relative to the PBS control midbrains; *n* = 3 for PBS and *n* = 4 for MPTP and MPTP+BMDCs. Fold change was determined using SA Bioscience software. **b** Ingenuity Pathway Analysis was used to determine the expression of genes associated with the inflammatory response in BMDCs + MPTP midbrain compared to MPTP control mice

To further assess the effects of BMDC-mediated suppression of neuroinflammation, we tested proinflammatory gene expression from midbrains of mice treated with PBS, MPTP, or BMDCs and MPTP. Gene expression was compared relative to midbrains from PBS controls. MPTP intoxication increased the expression more than 2-fold of *Ccl3, Cxcl10, Ccl4, Il1r1, Il1rn, Cebpb, Tlr2,* and *Csf1,* and down-regulated by more than 2-fold *C3ar1, Myd88, Tlr1, Ccl2, Ccr3. Ccr1, Tlr9, Ccl11, Cxcr4, Ccl25, Ccl12, Ripk2*, and *Tlr3* (Fig. 7a). Treatment with BMDCs by adoptive transfer and MPTP, increased by almost 2-fold the expression of *Il1rn*, the antagonist for IL-1 receptor [50]. Of the genes downregulated by MPTP, expression of *Ccl2, Ccl12, Ripk2,* and *Tlr3* was increased in BMDC-treated mice. In addition, adoptive transfer of BMDCs increased by more than 2-fold the proinflammatory genes *Il6ra, Il17a, Tnf,* and *Il6* and anti-inflammatory *Il10* gene. BMDCs also increased the expression of chemokine-related genes *Cxcl2, Cxcr2, Ccl1, Cxcl1, Cxcr1, Cxcl3, Cxcl9, Ccr4, Ccr2, Ccl20, Ccl24, Cxcl5,* and *Ccl8*. To better identify which genes are changed, midbrains of mice treated with BMDCs then MPTP intoxicated were compared to genes expressed in midbrains of mice treated with MPTP alone. Ingenuity Pathway Analysis showed 24 genes that expressions were changed by 2-fold or more and were associated with inflammatory response (Fig. 7b). The pattern of expression was indicative of inflammation as indicated by increases in *Tnf, Ccl11, Cxcl10, Cxcl6, Cxcr4, Ccr1, and Ccr3*, and genes related to pathogen recognition such as *Tlr1, Tlr2, Tlr3, Tlr9, Myd88*, and *Ripk2*. Decreased expression of *Il1r1, Cebpb, Ccl4*, and *Ccl3l3,* and increased expression of *Ccl2, Ccl24*, and *Il1rn* were indicative of an anti-inflammatory profile.

### BMDCs induce Tregs after MPTP intoxication

**Fig. 8:**
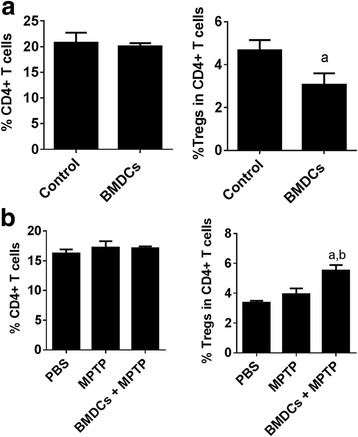
Acute inflammation induces Tregs after adoptive transfer of tolerogenic BMDCs. GM-CSF-generated BMDCs were cultured in media, harvested, and 1.5 × 10^6^ in 0.25 mls were adoptively transferred i.v. at each of 2 weekly transfers and the frequencies of splenic CD4+ cells and CD4+CD25+Foxp3+ Tregs were evaluated by flow cytometric analysis. Gates were drawn to include > 98% of the total lymphocytes or > 98% of CD4+ T cells. **a** Splenic T cell frequencies were determined seven days after the second transfer of BMDCs to naïve recipients. Control mice received no BMDCs. Means ± SEM were determined for control (*n* = 11) and BMDC-transferred mice (*n* = 17) and were evaluated by Student’s two-tailed t-test whereby *p* ≤ 0.05 compared to ^a^control mice. **b** After the second transfer of BMDCs, mice were intoxicated with four 16 mg/kg doses of MPTP, MPTP alone, or PBS alone. T cell frequencies were determined two days after intoxication. Means ± SEM were determined for *n* = 5 mice per each group and evaluated by one-way ANOVA followed by Tukey’s post hoc test whereby *p* ≤ 0.05 compared to mice treated with ^a^PBS alone or ^b^MPTP alone

We and others showed that in models of PD, GM-CSF is neuroprotective and that it increases Treg frequency and number as well as Treg activity in both PD and PD Models [35, 36, 51]. We thus assessed the effects of BMDCs to increase splenic Treg frequency in naïve mice and under neurodegenerative conditions. Seven days after serial weekly adoptive transfers of GM-CSF-derived BMDCs, flow cytometric analysis showed no change in overall CD4+ T cells, but showed decreased frequency of CD4+CD25+Foxp3+ Tregs (Fig. 8a). These results somewhat contrasted other results showing in vitro induction of Tregs by GM-CSF-matured BMDCs, either in the presence or absence of N-α-Syn (Fig. 4b) and GM-CSF-induced Tregs in vivo in the context of ongoing immune responses, disease, or MPTP intoxication [30, 35, 36]. We therefore tested whether ongoing inflammation was required to initiate BMDC-mediated Treg responses. After two serial adoptive transfers of BMDCs and MPTP intoxication, T cell and Treg frequencies were assessed in spleens 2 days after MPTP intoxication [46, 49]. While no changes in CD4+ T cell frequencies were detected among any treatment group, Treg frequency increased in mice treated with BMDCs prior to MPTP intoxication compared to PBS and MPTP controls (Fig. 8b). These data demonstrated that inflammatory signals are required for BMDC-mediated Treg induction or expansion. Additionally, that the frequency of CD4+ cells is not changed suggests that BMDCs do not promote Treg proliferation, but rather induce the differentiation of existing CD4+ cells into Tregs.

## Discussion

We and others showed that GM-CSF increases Treg numbers and function [28, 30, 35]. In models of neurodegeneration such as PD, Alzheimer’s disease (AD), traumatic brain injury (TBI), and stroke, administration of GM-CSF has been shown to be neuroprotective [36, 52]. Additionally, adoptive transfer of Tregs from naïve or GM-CSF treated donors is neuroprotective [35]. In a recent phase 1 clinical trial, treatment of PD patients with human recombinant GM-CSF (sargramostim) increased Treg numbers and Treg-mediated suppression of CD3/CD28-induced proliferation compared to placebo-treated PD patients [36]. Moreover, sargramostim improved motor deficits and initiation of motor activity as determined by diminished UPDRS, III scores and β-ERD signals by magnetoencephalography. These data underscore the potential for immune transformation by GM-CSF to diminish neurodegeneration in PD.

As T cells express few, if any, GM-CSF receptors, intermediaries are needed to affect Treg function. Several possible intermediary cell types include dendritic cells (DCs), macrophages, microglia, astrocytes, or myeloid-derived suppressor cells [53–57]. Based on recent data supporting the involvement of tolerogenic DCs in regulation of Treg activity, we chose to first examine the effects of GM-CSF on DCs to combat neuroinflammation and protect dopaminergic neurons. We also assessed the effects on DC-induced Treg formation by proteins that are oxidatively-modified and/or misfolded in PD, such as N-α-Syn.

First, bone marrow cells propagated in GM-CSF produce CD11b+CD11c+ cells with low expression of MHC II and CD86. The relative lack of cytokines and chemokines and relatively high levels of CD39 and CD73 associated with purinergic signaling that is important for switching immune cells to anti-inflammatory phenotypes, suggested these cells were of an immature DC phenotype and putatively tolerogenic capable of inducing Tregs [41]. To test that possibility, we found that co-culture of BMDCs with CD4+ T cells induced T cells with a CD4+CD25^hi^Foxp3+ Treg phenotype. Additionally, those phenotypic Tregs were shown to have Treg function as they inhibited CD3/CD28-induced proliferation of CD4+CD25- Tresp co-cultures in a dose-dependent fashion. Moreover, the function of BMDC-induced Tregs was significantly greater than that observed from fresh splenic isolates, and supported the tolerogenic capacity of the BMDCs. While GM-CSF can yield a heterogeneous mixture of myeloid cells, including BMDCs and macrophages, the tolerogenic capability to induce Tregs has not been ascribed to GM-CSF-generated macrophages [58, 59], thus the BMDCs described herein are congruent with prior results that demonstrate Treg-inducing capacity by tolerogenic DCs [31, 60]. Notably, tolerogenic BMDCs further exposed to media, GM-CSF, N-α-Syn, or both GM-CSF and N-α-Syn were capable of inducing significant increases in Treg numbers compared to starting isolates of primary CD4+CD25- T cells or those stimulated via CD3/CD28.

In clinical trials of GM-CSF in PD, Crohn’s Disease, or AD [36, 61, 62] (NCT01409915), treatment regimens use extended or chronic GM-CSF administration to maintain selective pressure on the anti-inflammatory response or Treg induction and function. In our model of extended exposure and/or proinflammatory stimulation, tolerogenic BMDCs were cultured in the presence of media or GM-CSF alone and remained unstimulated or were stimulated with N-α-Syn, an acute proinflammatory stimulus. Compared to media alone, continued culture in GM-CSF yielded few significant effects on surface expression of co-stimulatory molecules or expression of cytokine and chemokine genes and proteins. The few effects included increased expression of Jag-1, IL-10, an anti-inflammatory cytokine released from tolerogenic DCs [31, 39, 40], and IL-9 as well as decreased production of MIG (CXCL9) and MIP2 (CXCL2), suggesting that continued exposure to GM-CSF may maintain the tolerogenic state, particularly in light of increased Jag-1 which has been shown necessary for Treg induction [33, 34, 63]. However, the tolerogenic capacity for inducing Tregs was significantly diminished by 45% indicating that continued exposure to GM-CSF may eventually lessen the tolerogenic capacity and reduce Treg production.

N-α-Syn is an oxidatively modified and readily misfolded protein found in neuronal inclusions and extraneuronal environments of the brain in patients with PD, Lewy body disease, and multiple system atrophy [64, 65], and is also detected in the cervical lymph nodes by 20 h after MPTP intoxication [18]. Misfolded N-α-Syn is known to activate microglia as well as other myeloid and APCs of the innate immune system [18, 66]. While the function of native α-Syn remains largely unknown, modified or misfolded α-Syn can serve as a neoantigen. Thus, N-α-Syn activation of APCs that process and present modified epitopes of α-Syn and transmit proinflammatory signals during T cell activation, would support breaking or evasion of immunological tolerance leading to induction of effector T cells and antibody responses to the modified self-protein [18, 66]. Further, tolerogenic signals with or without modified α-Syn not only play a role in Treg induction, but also may influence the emergence of M1/M2 microglia phenotypes [67].

To assess the effect of N-α-Syn on phenotype and function, tolerogenic BMDCs were stimulated with N-α-Syn after culture in media or GM-CSF. Stimulation with N-α-Syn showed significant responses as determined by phenotype and expression of cytokines and chemokines regardless of extended culture in media or GM-CSF. Compared to non-stimulated BMDCs, stimulation with N-α-Syn increased surface expression of MHC II, CD86, and Jag-1 co-stimulatory molecules and genes for the maturation markers *Cd40, Cebpb*, and *Ccr7.* Additionally, proinflammatory gene expression and protein secretion were increased for IL-1β, IL-23α, IL-6, IFN-γ, TNF-α, IP-10 (CXCL10), LIX (CXCL5), MIG (CXCL9), MIP2 (CXCL2), and RANTES (CCL5). Together these data show that N-α-Syn stimulation drives BMDC pathways to a proinflammatory profile and suggests that differentiation might favor production of mature type 1 DCs that preferentially lead to induction of effector T cells, such as Th1 or Th17. Unexpectedly, co-culture of N-α-Syn-stimulated BMDCs with CD4+ T cells induced levels of Tregs that approached those attained upon culture in media alone, and significantly greater than those after culture GM-CSF alone. The possibility exists that acute or intense inflammatory responses may provide yet another signal or mechanism by which tolerogenic BMDCs induce conversion of conventional T cells to Tregs despite an otherwise proinflammatory environment.

Continued culture of BMDCs with GM-CSF followed by N-α-Syn stimulation diminished several phenotypic and functional responses, but not others. Of note, surface expression of CD86 and MHC II were diminished, but Jag-1 was increased to the highest levels of all culture conditions. Expression of proinflammatory cytokine and chemokine genes by BMDCs were overall elevated compared to culture in media alone, though were slightly diminished compared to those stimulated with N-α-Syn alone. More importantly, production and secretion of proinflammatory proteins were significantly increased over levels induced by N-α-Syn alone included IL-1α, IL-1β, IL-2, IL-5, IL-9, IL-12p70, and IL-17, but decreased LIX (CXCL5), MIG (CXCL9). Few anti-inflammatory mediators, such as IL-10, were increased. Together, these data suggest that BMDCs in the presence of GM-CSF and N-α-Syn induce an overall proinflammatory environment and may not be conducive for Treg development. Indeed, co-culture of CD4+ T cells with BMDCs previously treated with GM-CSF and stimulated with N-α-Syn, proved to induce the least number of Tregs with the exception of stimulating with anti-CD3/CD28 beads alone.

Overall, GM-CSF-induced BMDCs acquire an immature phenotype and exhibit a high degree of tolerogenic activity based on the ability to induce Tregs. That activity is maintained even after stimulation with a proinflammatory stimulus such as N-α-Syn and an elevated proinflammatory state. However, continued exposure to GM-CSF and possibly other mediators, result in a semi-mature state with diminished tolerogenic capability regardless of stimulation status. Both semi-mature and tolerogenic DCs have the ability to support transformation of CD4+ T cells to become Tregs [53], but at different levels depending on the state of activation. Indeed, while Treg induction capacity was least robust with semi-mature BMDCs produced by culture with GM-CSF and stimulated with N-α-Syn, levels of Tregs inducted were significantly greater than those within the initial isolation of CD4+ T cells or after CD3/CD28 stimulation. Previous work demonstrated that Jag-1 and OX40L are necessary for BMDC induction of Tregs [33]. However, under the conditions tested, no changes in the surface expression of OX40L were detected. This would suggest that expression of OX40L at the reported levels is sufficient to support Treg induction. Continued culture of tolerogenic BMDCs in GM-CSF increases surface expression of Jag-1, but diminishes their capacity to induce Tregs. This seemingly contrasts reports that Treg induction is dependent on Jag-1 dose and Notch interactions as determined by blocking antibodies or notch-signaling inhibitors [33]. Our data indicate that increases in Jag-1 surface expression were not sufficient to promote additional Treg induction, but were indirectly correlated (Pearson *r* = 0.55, *p* = 0.06, F = 4.42, DFn = 1.0, DFd = 10.0). This suggests that i) Jag-1 expression, albeit at low levels, is sufficient to promote Treg induction, ii) Jag-1 expression beyond a specific threshold provides negative signaling to inhibit Treg induction, or iii) other BMDC cell surface or internal signaling interactions are involved.

In PD models, administration of GM-CSF increases Treg number and function, attenuates inflammation in the brain, and protects dopaminergic neurons along the nigrostriatal axis [35, 51]. In light of our results showing Treg induction mediated by BMDCs, we tested whether tolerogenic BMDCs could also serve as another mechanism to provide protection from MPTP-mediated neurodegeneration. Indeed, adoptive transfer of GM-CSF-induced BMDCs prior to MPTP intoxication attenuated neuroinflammation and protected dopaminergic neurons along the nigrostriatal axis. Most interestingly, gene expression of proinflammatory mediators in the midbrain were overall increased compared to those from midbrains of mice treated with MPTP alone. These data suggested that adoptive transfer of BMDCs alter the chemokine environment, which in turn may change immune cell recruitment in brain. This is consistent with changes in chemokine gene expression in the substantia nigra of GM-CSF-pretreated mice [35]. However, gene changes also are indicative of decreased inflammation with down-regulation of *Il1r1*, the gene encoding the receptor for IL-1α and IL-1β which transduces signals intracellularly [68], *Cebpb*, a transcription factor which induces TNF expression [69], and chemokine genes *Ccl3l3* and *Ccl4*, which promote inflammation [70] and leukocyte recruitment into the brain [71]. *Ccl24* encodes a chemokine released from M2 macrophages which can recruit T cells, especially Tregs [72, 73]. In glioblastoma multiforme, *Ccl2* is important for Treg recruitment into the brain and may perform a similar function after MPTP intoxication [74, 75]. *Il1rn* prevents the recruitment of IL-1 receptor associated protein to IL-1 receptor which is required for transduction of signals within the cell [76], therefore its increased expression, combined with downregulation of *Il1r1* would diminish IL-1α and IL-1β signaling. Together with results showing diminished reactive microglia in Fig. 6, further support the notion that BMDCs diminish neuroinflammation.

As GM-CSF increases Treg numbers [35], we assessed the ability of tolerogenic BMDCs to induce Tregs after adoptive transfer. Unexpectedly, in naïve mice, Treg frequencies diminished after transfer. The causes for these results remain unclear, but may be in part due to limited numbers of BMDCs (1.5 × 10^6^) that could be safely transferred. Another possibility is that without inflammatory insult, induction of Tregs may be inactive or suppressed. Our results suggested that BMDCs may not function by the same mechanism(s) as GM-CSF or that GM-CSF has effects independent of DC-mediated Treg induction. The possibility that GM-CSF or BMDCs are protective apart from the induction of Tregs is also suggested. Indeed, GM-CSF can directly promote the survival of PC12 neurons and primary neurons from MPP+ neurotoxicity [51] and could increase other regulatory immune cells such as myeloid-derived suppressor cells (MDSCs) [56]. Interestingly, the possibility that endogenous induction of Tregs by BMDCs within the bone marrow cannot be ruled out as a small population (< 0.5%) of CD4+CD25+Foxp3+ Tregs constitute the bone marrow stem cell population which was shown to be neuroprotective in a rat model of experimental stroke [52]. Such alternative mechanisms should be examined in future studies. Because the adoptive transfer of BMDCs was protective in the MPTP model, it may be possible that the adoptive transfer of tolerogenic bone marrow-derived or monocyte-derived dendritic cells may promote Treg induction and decrease PD-associated motor symptoms. It may be possible to induce, expand or prime autologous DCs ex vivo for adoptive transfer as a personalized therapeutic modality to elicit clinical benefits.

## Conclusion

GM-CSF-induced BMDCs have tolerogenic properties that culminate in the transformation of conventional CD4+ T cells to Tregs. These exhibit higher levels of activity than Tregs from fresh spleen isolates. Continued culture of BMDCs in GM-CSF and/or stimulation with N-α-Syn diminishes the capability to transform Tregs. Continued exposure to GM-CSF reduces the BMDC capability to transform T cells to Tregs. Adoptive transfer of tolerogenic BMDCs attenuated neuroinflammation as determined by reduced numbers of reactive microglia and protected dopaminergic neurons and their striatal termini from MPTP-induced neurodegeneration. In contrast to in vitro results whereby tolerogenic BMDCs readily convert conventional T cells to Tregs, adoptive transfer of BMDCs to naïve animals diminished numbers of splenic Tregs; however, acute inflammatory signals by MPTP-intoxication after adoptive transfer of BMDCs significantly increased Treg numbers. Together, these data demonstrate a role for DCs in modulating inflammation and subsequent neurodegeneration. The results also provide another mechanism by which GM-CSF modulates innate immune function to further regulate adaptive immunity and provide neuroprotection. Moreover, administration of GM-CSF or other agents that induce tolerogenic DCs leading to increases in numbers of Tregs and Treg activity, present an attractive therapeutic strategy for decreasing neuroinflammation in autoimmune or neurodegenerative diseases.

## Abbreviations

APCs: Antigen-presenting cells
BMDCs: Bone marrow-derived dendritic cells
BSA: Bovine serum albumin
CFSE: Carboxyfluorescein succinimidyl ester
DAB: 3,3′-diaminobenzidine
DCs: Dendritic cells
DPBS: Dulbecco’s phosphate-buffered saline
FSB: Flow stain buffer
GM-CSF: Granulocyte-macrophage colony-stimulating factor
HBSS: Hanks’ balanced salt solution
i.v.: Intravenous
MDSCs: Myeloid-derived suppressor cells
MFI: Mean fluorescent intensity
MPTP: 1-methyl-4-phenyl-1,2,3,6-tetrahydropyridine
N-α-Syn: nitrated alpha synuclein
PBS: Phosphate-buffered saline
PD: Parkinson’s disease
RBCs: Red blood cells
s.c.: subcutaneous
TBI: Traumatic brain injury
Teffs: Effector T cells
TH: Tyrosine hydroxylase
TLRs: Toll-like receptors
Tregs: Regulatory T cells
Tresps: Responder T cells
UPDRS: Unified Parkinson’s disease rating scale
